# Supplementary figures and images for: The complete chloroplast genome sequences of eight Orostachys species: Comparative analysis and assessment of phylogenetic relationships
Source: PLoS One. 2022 Nov 10;17(11):e0277486. doi: 10.1371/journal.pone.0277486 (PMC9648774; doi:10.1371/journal.pone.0277486)

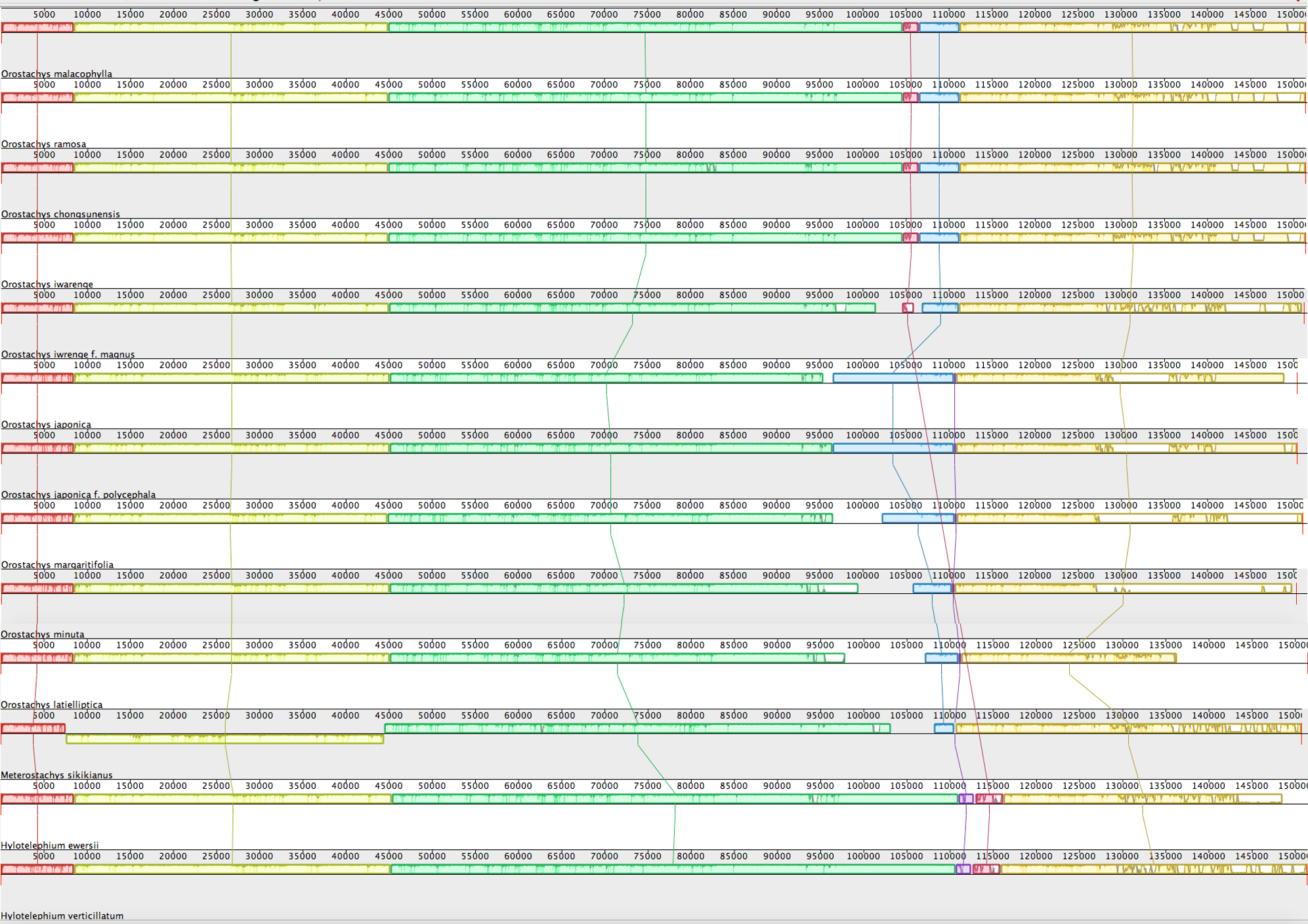

Supplement: S1 Fig — (PNG) [file pone.0277486.s001.png]
