# Supplementary material for: The complete chloroplast genome sequences of eight Orostachys species: Comparative analysis and assessment of phylogenetic relationships
Source: PLoS One. 2022 Nov 10;17(11):e0277486. doi: 10.1371/journal.pone.0277486 (PMC9648774; doi:10.1371/journal.pone.0277486)
Supplement: Attachment — Submitted filename: Response to Reviewers.docx [file pone.0277486.s004.docx]

**Response to Reviewers**

We are pleased to resubmit for publication the revised version of PONE-D-22-22176 “**The complete chloroplast genome sequences of eight *Orostachys* species: comparative analysis and assessment of phylogenetic relationships**” We appreciated the constructive criticisms of the reviewers. We have addressed each of their concerns as outlined below.

**Journal requirements:**

**1. Please ensure that your manuscript meets PLOS ONE's style requirements, including those for file naming. The PLOS ONE style templates can be found at**

https://journals.plos.org/plosone/s/file?id=wjVg/PLOSOne_formatting_sample_main_body.pdf and

https://journals.plos.org/plosone/s/file?id=ba62/PLOSOne_formatting_sample_title_authors_affiliations.pdf

*→ We checked and reflected the template style of PLOS ONE at the request of the journal.*

**2. Please ensure that you refer to Figure 4 in your text as, if accepted, production will need this reference to link the reader to the figure.**

*→ Referenced Figure 4 in the manuscript.*

**3. Please review your reference list to ensure that it is complete and correct. If you have cited papers that have been retracted, please include the rationale for doing so in the manuscript text, or remove these references and replace them with relevant current references. Any changes to the reference list should be mentioned in the rebuttal letter that accompanies your revised manuscript. If you need to cite a retracted article, indicate the article’s retracted status in the References list and also include a citation and full reference for the retraction notice.**

*→ References have been reviewed and revised once again.*

**Reviewer 1.**

**This is a well-written manuscript. I suggest a minor revision for this ms. In the Nucleotide diversity among the Orostachys, pls add the result of rbcL and matK. Since rbcL and matK are the core DNA barcodes, both sequences could be retrieved from the chloroplast genome. It will be helpful to provide the relevant information. And I encourage the authors to submit their voucher photos and the sequences of rbcL and matK to the BOLD system, to facilitate the widespread use of their study results.**

*→ We added the result of nucleotide diversity of rbcL and matK. We are trying to upload the sequences of rbcL and matK to the BOLD system, but it is not completed at this time. We promise to upload as soon as possible.*

**Reviewer 2.**

**This study analyzed the complete chloroplast genomes of eight *Orostachys* species and compared these sequences with published sequences of the chloroplast genomes of the congeneric and closely related genera *Meterostachys* and *Hylotelephium* to assess the phylogenetic relationships between the two subfamilies of *Orostachys* and its closely related genera. To some extent, the taxonomic status of several taxa is clarified and the fact that taxonomists have different opinions on the classification of certain species is resolved. The techniques used in the study are reliable and the data support its conclusions, especially the phylogenetic analysis gives reliable results, and also well presented the shortcomings of the study and the direction of subsequent research, but the content part of the analysis of the chloroplast genome could be more complete.**

**In lines 314-315, “In our experience, branching at the base of the stem is an occasional mutation in *O. malacophylla*.” It is suggested here that it might be more rigorous to find literature or evidence for this rather than relying on experience, or it might be possible to describe clearly what experience is relied on，from which I think it is not very rigorous to conclude that *O. malacophylla*, *O. ramosa*, and *O. chongsunensis* are the same species.**

*→ We have added photos (Fig 6) to support our experience.*

**Similarly, in lines 316-317, “the purplish variegate leaves of O. chongsunensis are thought to be due to the growth environment” providing the relevant basis here would make the conclusion more convincing.**

*→ We have also added photos (Fig 6) to support this.*

**It is strongly suggested to provide photographs of the studied species, especially those of the representative ones of each concerned group, for the readers’ easier understanding.**

*→ We have added various photos (Fig 6) related to this study.*
